# Inorganic nitrate as a treatment for acute heart failure: a protocol for a single center, randomized, double-blind, placebo-controlled pilot and feasibility study

**DOI:** 10.1186/s12967-017-1271-z

**Published:** 2017-08-08

**Authors:** Roman Falls, Michael Seman, Sabine Braat, Joshua Sortino, Jason D. Allen, Christopher J. Neil

**Affiliations:** 1 Western Centre for Health Research and Education, Western Health, Melbourne, Australia; 2 Department of Medicine-Western Health, Melbourne Medical School, The University of Melbourne, Melbourne, Australia; 3 Clinical Exercise Science Research Program, Institute of Sport Exercise and Active Living (ISEAL), Melbourne, Australia; 4 Melbourne School of Population and Global Health and Melbourne Clinical and Translational Sciences Platform (MCATS), Parkville, Australia; 5 Western Health Cardiology, Footscray Hospital, Gordon St, Locked Bag 2, Footscray, VIC 3011 Australia

**Keywords:** Heart failure, Acute heart failure, Acute decompensated heart failure, Sodium nitrate, Nitric oxide, Non-invasive cardiovascular monitoring

## Abstract

**Background:**

Acute heart failure (AHF) is a frequent reason for hospitalization worldwide and effective treatment options are limited. It is known that AHF is a condition characterized by impaired vasorelaxation, together with reduced nitric oxide (NO) bioavailability, an endogenous vasodilatory compound. Supplementation of inorganic sodium nitrate (NaNO_3_) is an indirect dietary source of NO, through bioconversion. It is proposed that oral sodium nitrate will favorably affect levels of circulating NO precursors (nitrate and nitrite) in AHF patients, resulting in reduced systemic vascular resistance, without significant hypotension.

**Methods and outcomes:**

We propose a single center, randomized, double-blind, placebo-controlled pilot trial, evaluating the feasibility of sodium nitrate as a treatment for AHF. The primary hypothesis that sodium nitrate treatment will result in increased systemic levels of nitric oxide pre-cursors (nitrate and nitrite) in plasma, in parallel with improved vasorelaxation, as assessed by non-invasively derived systemic vascular resistance index. Additional surrogate measures relevant to the known pathophysiology of AHF will be obtained in order to assess clinical effect on dyspnea and renal function.

**Discussion:**

The results of this study will provide evidence of the feasibility of this novel approach and will be of interest to the heart failure community. This trial may inform a larger study.

## Background and rationale

### Acute heart failure: incidence and current treatment

In the United States, acute heart failure (AHF), also known as acute decompensated heart failure, is responsible for over 1 million hospital admissions annually [1] and is the leading reason for hospitalization among patients over 65 years of age. AHF refers to the heterogeneous syndrome of the gradual or rapid onset of worsening signs and/or symptoms of heart failure [2, 3], attributed to fluid overload, raised ventricular filling pressures and/or pulmonary congestion [4].

Although contemporary medical and device-based therapies (including non-invasive ventilation and ventricular assist devices) have substantially improved the outlook for patients with *chronic* heart failure, the lack of established effective treatment strategies for patients presenting with AHF has been identified as a major need [5]. This, together with (i) the re-emerging role of vasodilators in AHF, (ii) evidence for acutely impaired nitric oxide (NO) bioavailability and (iii) the potential therapeutic benefit of inorganic sodium nitrate, will be discussed as background to this protocol.

### Vascular resistance in the hemodynamics of AHF

A discussion of typical hemodynamic profiles in the spectrum of AHF presentations is crucial to understand the re-emerging role of vasodilators in this context. Patients with AHF generally present with an elevated pulmonary capillary wedge pressure (PCWP), resulting in congestion (interstitial and/or alveolar pulmonary edema), present in the majority of cases of AHF. On the other hand, cardiac output/cardiac index (CI) can vary substantially within the AHF population. The majority of AHF patients (69%) actually present with a CI in the normal range, as illustrated by the work of Nohria et al. [6]. Furthermore, the majority of patients with AHF are known to present with hypertension (50%), rather than normotension (47%) or hypotension (3%) [7]. By inference, given that such patients are typically not hyperdynamic, systemic vascular resistance (SVR) is likely to be generally and markedly elevated at the time of acute presentations in this group. Elevated SVR was found to be the most consistent hemodynamic abnormality in a cross-sectional study employing non-invasive cardiac output measurements in a large cohort of AHF patients [8].

Acute elevation of SVR has several implications in the context of AHF. Increased SVR would generally be attributed to impaired vasorelaxation (or failure of vasodilation) at an arteriolar level, as a net result of neuroendocrine and paracrine signals, in addition to the influence of vasoactive drugs. Importantly, activation of the sympathetic nervous system, with arteriolar vasoconstriction and reduced venous capacitance, is thought to promote the centralization of blood volume, in the genesis of pulmonary congestion [9, 10]. The effects of high SVR on afterload are also likely to be crucial in the pathophysiology of AHF, which has been conceptualized as a condition of afterload mismatch, as reviewed by Cotter et al. [8, 11]. In respect to afterload, it should be noted that there are pulsatile and non-pulsatile elements of afterload and both are known to be abnormal in AHF [12, 13]. All of the above considerations are components of an emerging vasculocentric model of AHF [14].

### Current therapeutics in AHF

The primary therapeutic objective during AHF hospitalization is decongestion, whilst avoiding the complications of hypotension and worsening renal function (WRF). Currently, loop diuretic is universally recommended in international guidelines, with the directed use of noninvasive ventilation, nitrovasodilators and inotropic support, in more severe cases [3, 15]. With the exception of noninvasive ventilation, this basic therapeutic tool kit has been consistent for over 30 years. Unfortunately, despite substantial effort, no novel pharmacological strategy has thus far been shown to be superior. Positive inotropes have been associated with increased mortality [16]. In regards to high dose intravenous organic nitrates (e.g. isosorbide dinitrate, glyceryltrinitrate), despite their availability and apparent benefit in several relatively small phase II studies [17–19], use is hampered by the development of tolerance [20], as well as the generation of reactive oxygen species [21] and impaired biotransformation in heart failure patients [22].

Several novel neurohumoral therapeutic strategies, including recombinant B-type natriuretic peptide infusion, adenosine receptor antagonism and vasopressin receptor antagonism, have not demonstrated an advantage over standard therapy [23–25]. Mechanical diuresis via ultrafiltration has likewise not demonstrated superiority to standard medical diuresis, in fact demonstrating an increased incidence of adverse events [26]. More recently, serelaxin, a recombinant vasodilating peptide, has shown some promise in a study by Teerlink et al. [27]. Whilst there was a significant benefit seen in dyspnea relief during treatment and biomarker endpoints, the 30-day readmission rate was not reduced. Intriguingly, however, the mortality at 6 months was improved over placebo. This finding has focused interest on vasodilation in AHF, as well as informed a large phase III study [28].

### Nitric oxide bioavailability in AHF

The normal physiology of vascular relaxation involves the generation of NO via endothelial nitric oxide synthase, from l-arginine; NO freely diffuses to adjacent smooth muscle cells to activate soluble guanylate cyclase (sCG), which induces vasorelaxation via a second messenger cyclic guanylate monophosphate. Heart failure is associated with decreased NO bioavailability and dysfunction of endothelial NOS [29]. In acute decompensation, there is decreased overall NO production, as reflected by a 38% decrease in systemic nitrate and nitrite [30]. An additional mechanism by which NOS function may be acutely impaired is a substantial increase in levels of circulating asymmetric dimethylarginine (ADMA), an endogenous inhibitor of NOS, which was measured simultaneously with reduced nitrate and nitrite levels, by Saitoh and colleges [30]. Thus, in the AHF context, impaired NOS function may be at the nexus of poor NO biosynthesis/bioavailability and elevated SVR. Methods to therapeutically circumvent this deficiency may result in therapeutic benefit.

**Fig. 1:**
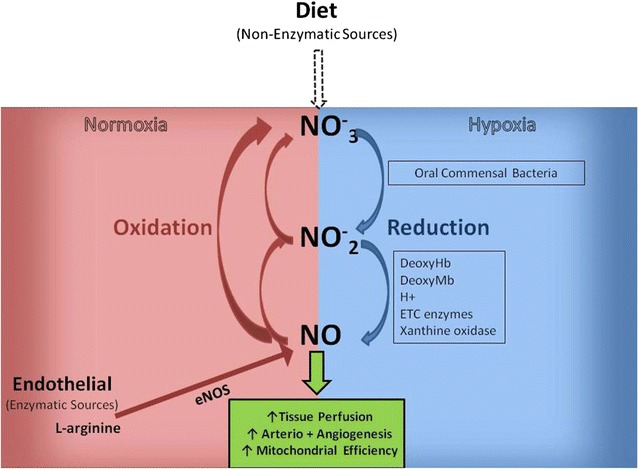
Nitrate–nitrite–nitric oxide formation/recycle pathways. In the presence of oxygen, endothelial nitric oxide synthase (NOS) catalyzes the oxidation l-arginine to nitric oxide (NO). NO is oxidized to nitrite (${\text{NO}}_{2}^{ - }$) and nitrate (${\text{NO}}_{3}^{ - }$). Dietary intake of inorganic nitrate (leafy green vegetables, beetroot) has been shown to increase plasma nitrate, which is concentrated in saliva where is it is reduced to nitrite by commensal oral bacteria. Nitrite can then be absorbed and further reduced to NO via several mechanisms, under hypoxic conditions. The pools of circulating nitrate and nitrite are subject to renal excretion, but are also able to be recycled back to NO [52] (Source: Allen et al. [52]. (Reproduced with permission from J.D Allen))

The concept of a circulating pool of inorganic nitrite (${\text{NO}}_{2}^{ - }$) and nitrate (${\text{NO}}_{3}^{ - }$) as an effective NO donor, independent of endothelial NOS function, has received increasing attention over the past decade [31]. Nitrite levels can be increased via direct administration (e.g. intravenous or inhaled), or by oral intake of nitrate (typically in foods or as a pure compound, with a half-life of 5–6 h), which is subsequently reduced in vivo, increasing plasma concentrations of nitrite [32] (see Fig. 1). The therapeutic potential of a nitrate/nitrite supplementation strategy in cardiovascular disease is underscored, when three additional facts are considered: (i) circulating nitrite may selectively donate NO to hypoxic vascular beds [33], (ii) repeat administration does not seem to induce tolerance [34], unlike the situation with organic nitrates and (iii) it is very well tolerated with modest effects on systemic blood pressure (e.g. dietary supplementation of inorganic nitrate produced an average blood pressure lowering of 8.1/3.8 mmHg [35]).

Furthermore, recent experience in stable heart failure patients suggests that inorganic nitrate/nitrite supplementation will be effective as a decongestive and vasorelaxant therapy in AHF. In stable *compensated* heart failure (NB. with *preserved* ejection fraction), Zamani et al. demonstrated in 17 patients following an acute dose of beetroot juice (containing 8.4 mmol inorganic nitrate), an increase in aerobic work capacity in a maximal supine cycle ergometer test, which was accompanied by a reduction in SVR, and an increase in cardiac output [36]. In advanced but compensated heart failure patients, the Frenneaux group recently reported on the central hemodynamic effects of a 5-min high dose infusion of sodium nitrite (NaNO_2_). At an infusion rate of 50 μg/kg/min, a 12% reduction in SVR was observed, together with a 13% increase in cardiac output and substantial venodilation, with only a 4 mmHg drop in systolic blood pressure [37]. Whilst these short infusions did not produce any methemoglobinemia, a study of prolonged infusions (up to 48 h) in healthy volunteers by Pluta et al. of ~5 μg/kg/min sodium nitrite did result in methemoglobinemia [38]. Thus, whilst intravenous sodium nitrite holds therapeutic promise for hemodynamic optimization in advanced HF, due to the limited number of studies, the safety of sustained treatment in an AHF episode currently remains a concern.

On the other hand, oral administration of inorganic sodium nitrate has the advantage of having been more widely studied. For example, supplementation of sodium nitrate in the range of 5.1–45 mmol/day (321–2790 mg) was demonstrated to be safe in a meta-analysis of studies in hypertensive patients [39] and may therefore be a safe means of delivering the hemodynamic benefits of NO in AHF. Furthermore, no clinically significant methemoglobinemia (defined by levels ≥5%) has been demonstrated to occur at this dose [40, 41].

## Methods and design

**Fig. 2:**
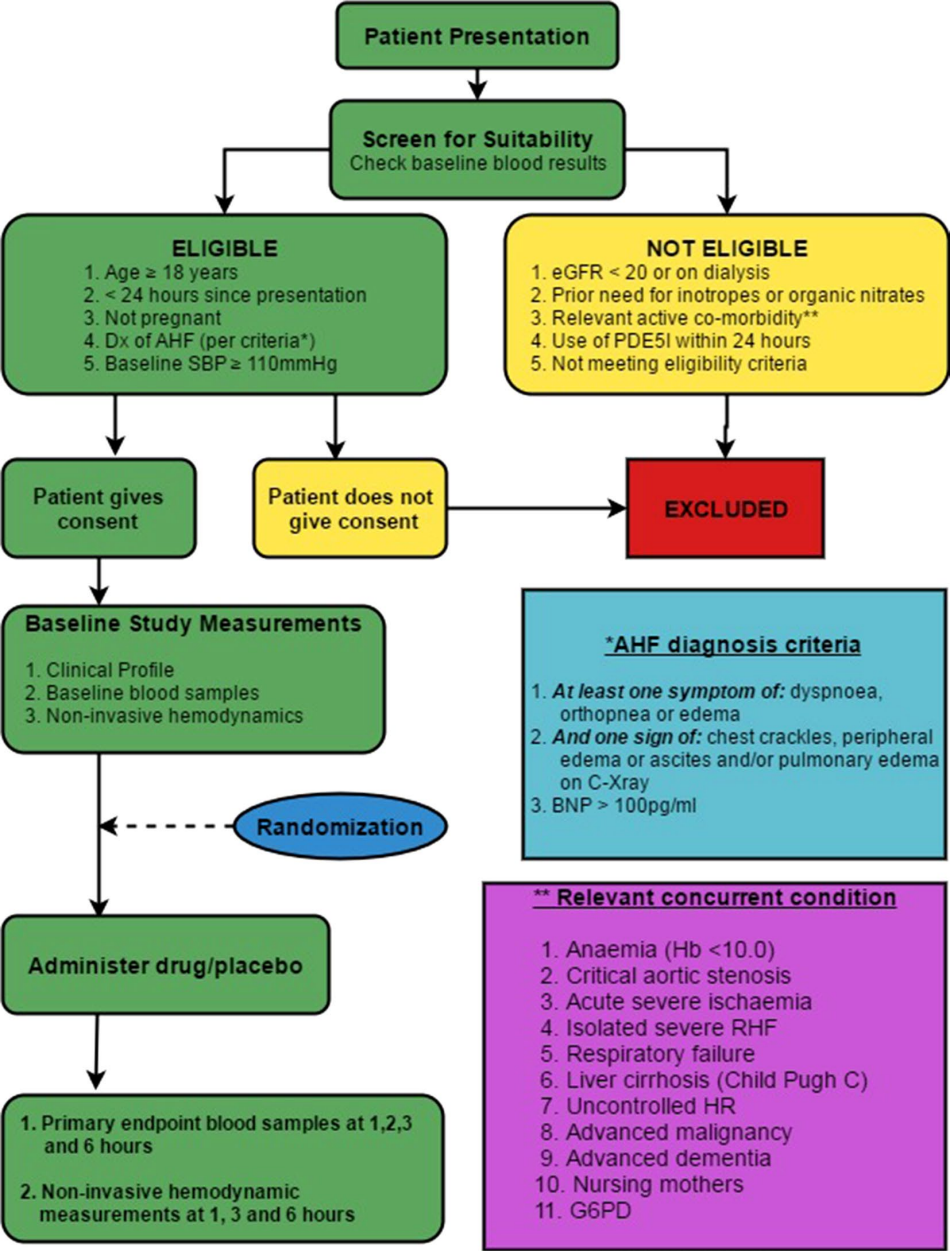
Overview of trial design

This study is considered a pilot and feasibility study [42]. It is a single-center, double blind, placebo-controlled, randomized trial evaluating inorganic sodium nitrate versus placebo. The trial will be conducted at two hospital campuses (Footscray Hospital and Sunshine Hospital) within the Western Health hospital network, in metropolitan Melbourne, Australia. The trial is funded by a grant from the University of Melbourne. The protocol conforms to the SPIRIT 2013 statement, and the CONSORT-EHEALTH checklist. An overview of the trial design is presented in Fig. 2. The trial is registered with the Australian New Zealand Clinical Trials Registry (ACTRN12616000951459).

### Aims and hypotheses

The aim of this study is to evaluate the feasibility of inorganic sodium nitrate administration, using a single dose (8.4 mmol) and to gain an indication of its effects as a vasoactive treatment for AHF, via bioconversion to NO precursors.

#### Primary hypothesis

Supplemental sodium nitrate in comparison to placebo will result in (a) increased plasma nitrite levels (as a surrogate for a circulating potential NO pool) in parallel with (b) improved vasorelaxation, as assessed by SVR.

#### Secondary hypotheses

Supplemental sodium nitrate in comparison to placebo will result in:

- reduced arterial stiffness, as assessed by augmentation pressure (AP) and augmentation index (AI);
- preserved/improved renal function, as assessed by serial plasma cystatin C levels;
- no clinically significant elevation of systemic methemoglobin levels, i.e. not exceeding 5%;
- greater dyspnea relief as per the Likert scale;
- greater systemic oxygenation as determined by arterial oxygen saturation as assessed by pulse oximetry;
- improved plasma troponin and B-type natriuretic peptide (BNP) concentrations;
- reduced hospital length of stay (LOS) and incidence of clinical deterioration and complications of treatment, including WRF, progression to respiratory failure/ventilatory support, hemodynamic compromise, stroke, and death.

### Subject recruitment

#### Inclusion criteria

Eligible subjects will be ≥18 years who present to hospital within the previous 24 h with AHF and underlying HF with reduced ejection fraction (HFrEF), diagnosed on the basis of at least one symptom (dyspnea, orthopnea or edema) and one sign (chest crackles, peripheral edema, ascites) and/or radiological confirmation of pulmonary vascular congestion on a chest X-ray (CXR). Both the Boston criteria [43] and plasma BNP concentrations (>100 pg/mL) will be used to independently confirm (or refute) the AHF diagnosis. The diagnosis of HFrEF will be based on the European Society of Cardiology Guidelines [3] and adjudicated by the study cardiologist (CN). In order to minimize the risk of serious hypotension in this study, it will be required that the patient must have a baseline systolic blood pressure of ≥110 mmHg at enrolment.

#### Exclusion criteria

Patients will be excluded if they have a pre-existing and ongoing need for either inotrope therapy or organic nitrates. Patients will be ineligible for enrollment if they have an estimated glomerular filtration rate (eGFR) <20 mL/min/1.73 m^2^ or are receiving renal replacement therapy (dialysis). The following concomitant conditions will represent further exclusion criteria: anemia with a hemoglobin of less than 10.0 g/dL, critical aortic stenosis, acute severe ischaemia, isolated severe right sided heart failure, respiratory failure, cirrhosis of the liver (Child–Pugh class C) and uncontrolled heart rate (tachycardia or unaddressed bradycardia), advanced malignancy or advanced dementia. Women of child-bearing potential or nursing mothers will also be excluded. Finally, those with phosphodiesterase 5 inhibitor (PDE5I) use within the preceding 24 h will be excluded, on specific enquiry.

#### Randomization protocol and blinding of treatment allocation

Following informed consent and all baseline assessments, eligible patients will be randomized 1:1 to sodium nitrate or placebo. The randomization list will be computer-generated by an independent statistician. A stratified block permuted randomization will be used with hospital campus as the stratification variable. The block size will not be disclosed to ensure concealment. Randomization will be enabled through a web-based application using REDCap^®^ electronic data capture tools hosted at the University of Melbourne. Patient enrollment and random assignment will be performed by trial investigators. Sodium nitrate and placebo will be packaged identically and labelled by the hospital pharmacy. Treatment assignment numbers will be logged with the hospital pharmacy. Aside from the trial’s hospital pharmacist, patients, nurses, treating clinicians, the trial investigators and the statistician will be blind to treatment allocation.

### Preparation and administration of the investigational product

In addition to standard care, patients will be randomly allocated to receive either a single dose of oral sodium nitrate (8.4 mmol, or 714 mg) or matching oral placebo. Food-grade sodium nitrate, a crystalline powder, will be compounded locally into white gelatin capsules. An identical placebo capsule will also be provided, as per randomization, containing an inert substance (microcrystalline cellulose, Avicel^®^).

### Techniques for outcome determination

#### Measurements for primary outcome determination

Measuring NO directly is inherently difficult, due to compound instability and short biological half-life. The majority of studies measure the concentration of plasma nitrite in order to evaluate the degree of bioconversion resulting from nitrate dosing. In this context, plasma nitrite is therefore a surrogate, reflecting the effective NO pool [44].

**Fig. 3:**
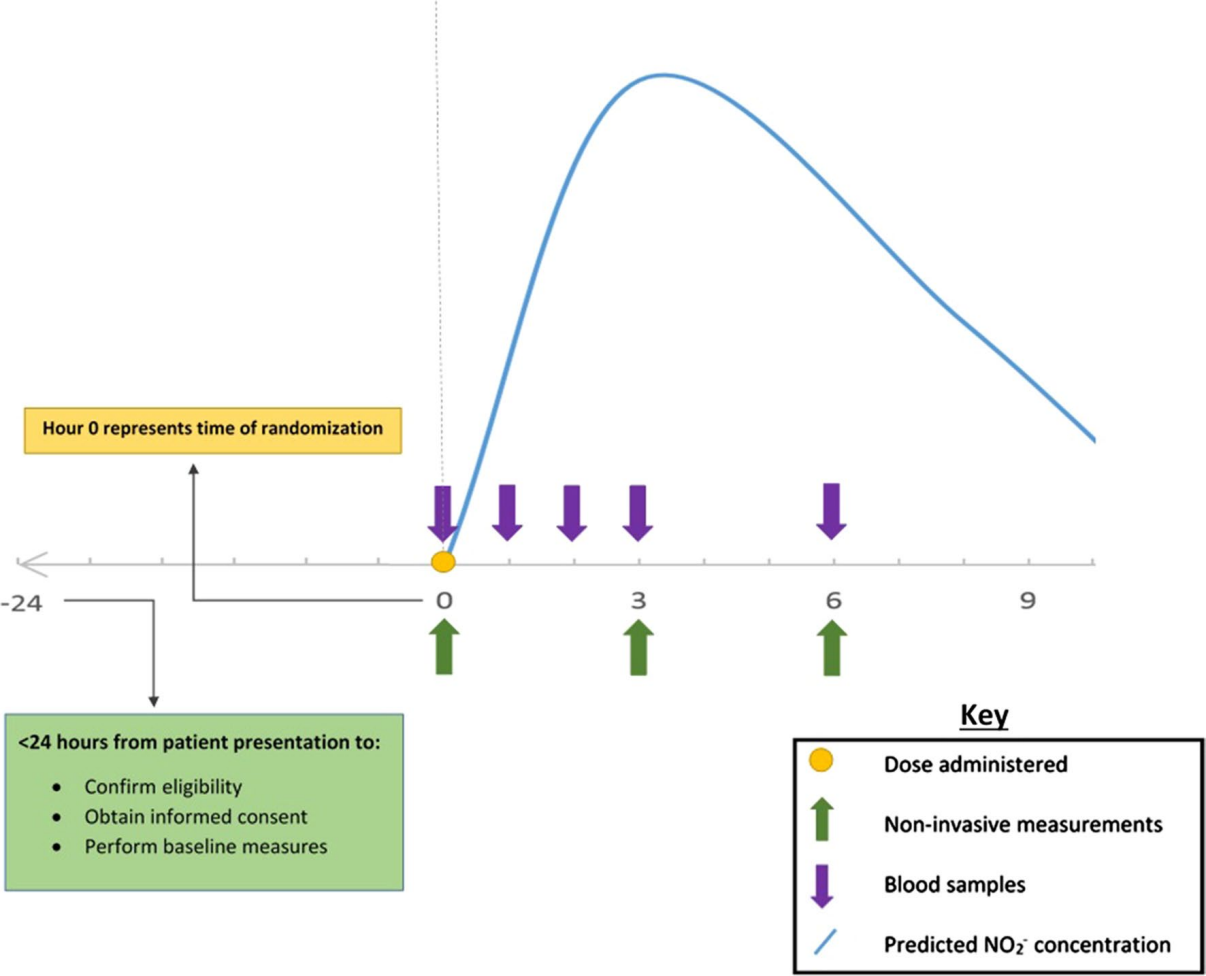
Overview of trial timeline in hours

In order to assess sodium nitrate bioconversion and pharmacokinetics (including steady state concentrations), blood sampling will occur at time points based on known peak plasma ${\text{NO}}_{3}^{ - }$ and ${\text{NO}}_{2}^{ - }$ concentrations and half-lives of orally administered inorganic nitrate [44, 45]. Blood will be drawn at 0, 1, 2, 3 and 6 h, centrifuged for plasma, snap frozen in liquid nitrogen, and stored at −80 °C until analysis. Subsequent batched NO analysis (NOA) will be performed to determine plasma nitrate and nitrite levels. NOA will be performed by chemical reduction, followed by chemiluminescence (Sievers NO analyzer) as previously described [44, 46]. An overview of the trial timeline is presented in Fig. 3.

In order to assess the vasomotor effects of supplemental nitrate, SVR (indexed to body surface area) will be serially assessed non-invasively. In order to obtain this, bioimpedance cardiography cardiac output measurement will be performed utilizing the PhysioFlow^®^ device, together with simultaneous blood pressure [47] at 0, 3 and 6 h.

#### Measurements for secondary outcome determination

- Indices of arterial stiffness (AP and AI) will be derived utilizing the SphygmoCor^®^ device as previously described [48, 49] at 0, 3 and 6 h.
- Venous methemoglobin will be assayed from blood drawn at time points 0, 3 and 6 utilizing a standard blood gas analyzer.
- *Blood tests:* plasma cystatin C, troponin and BNP will be determined at 0 and 6 h, and pre-discharge.
- *Clinical measurements:* subjective dyspnea and systemic oxygenation will be prospectively measured via the Likert scale and finger plethysmography, respectively at 0, 3 and 6 h; length of stay and the incidence of complications (WRF, progression to respiratory failure, hypotension/hemodynamic compromise, stroke, and death) will be obtained from the medical record at the time of patient discharge; WRF, or acute kidney injury, will be specifically quantified according to the Kidney Disease Improving Global Outcomes (KDIGO) classification.

### Data collection and data management

Study data will be collected and managed using REDCap^®^ electronic data capture tools hosted at the University of Melbourne [50]. REDCap^®^ (Research Electronic Data Capture) is a secure, web-based application designed to support data capture for research studies, providing (1) an intuitive interface for validated data entry; (2) audit trails for tracking data manipulation and export procedures; (3) automated export procedures for seamless data downloads to common statistical packages; and (4) procedures for importing data from external sources. Training of those who collect, check and enter study data will facilitate high quality data, including regular data checks for inconsistency and missing data between and within measurements. Before the start of the statistical analysis, a check will be performed to evaluate the correctness of the randomization.

### Statistical methods

In this pilot and feasibility study, the sample size calculation was limited by available resources. As there is currently no relevant literature available to inform the sample size calculation on the primary outcomes in this vulnerable patient population, a total of 40 acute heart failure patients was felt to be realistic and achievable. Twenty patients per treatment group will allow detection of a moderate effect size of 0.6 between sodium nitrate and placebo with a power of 85% using a *t* test to evaluate the change from baseline to a post-baseline time point at a two-sided significance level of 0.05, assuming a correlation of 0.8 between the baseline and post-baseline measurement. Based on the observed standard deviation for SVR of 296–416 (dyn s/cm^5^) at baseline in a total of 40 patients reported by Kim et al. [51] and assuming the standard deviation will be similar after sodium nitrate treatment, an effect size of 0.6 corresponds to an absolute treatment effect of about 178–250 (dyn s/cm^5^) in favour of sodium nitrate compared to placebo, which is considered a clinically relevant improvement.

All randomized patients will be included in the analysis of all outcomes. Descriptive statistics will be used to describe the characteristics of the sample and summarize the data by treatment group and overall. The primary outcomes plasma nitrite levels and SVR will be analyzed separately using a mixed effect repeated measures analysis, including in the model the factors treatment group, time point and treatment by time interaction, while also accounting for baseline plasma levels, baseline by time interaction and the factor hospital campus. The primary hypothesis will be evaluated by examining the main difference between sodium nitrate and placebo as well as the difference between both over time. Secondary outcomes measured will be analyzed similarly to the primary outcomes. If the underlying assumptions of the primary or secondary outcome data are violated, then either an appropriate transformation to allow for parametric methods or non-parametric (or bootstrapping) methods will be used. The number and percentage of patients with events of interest (e.g. complications) will be compared using the Fisher’s exact test. All tests will be two-sided.

## Discussion

The combined aims of this project seek to determine the effects of a single dose of orally administered *inorganic nitrate* in patients with AHF. While previous research indicates that administration of *organic nitrates,* such as glyceryltrinitrate, can be effective at improving the acute hemodynamic and clinical status of patients with AHF, the induction of tolerance limits usefulness in acute practice. In contrast, we have outlined the therapeutic potential of inorganic nitrate for the clinical context of AHF, which can be given orally and is not subject to the pharmacological tolerance, noting that there have been no recorded studies examining either the bioconversion of inorganic nitrate, or its vascular effects, in this context. If oral inorganic nitrate supplementation is shown to enhance NO bioavailability and produce beneficial changes in peripheral vascular function in AHF, appropriately powered studies may be undertaken. This mechanistic translational study, therefore, has the potential to provide the foundation of a new strategy in AHF, where therapeutic options are acknowledged to be limited.

## Abbreviations

ADMA: asymmetric dimethylarginine
AHF: acute heart failure
AI: augmentation index
AP: augmentation pressure
BNP: B-type natriuretic peptide
CI: cardiac index
CXR: chest X-ray
eGFR: estimated glomerular filtration rate
G6PD: glucose-6-phosphate dehydrogenase
Hb: hemoglobin
HR: heart rate
LOS: length of stay
NaNO_2_: sodium nitrite
NaNO_3_: sodium nitrate
NO: nitric oxide
$$ {\text{NO}}_{2}^{ - } $$: nitrite
$$ {\text{NO}}_{3}^{ - } $$: nitrate
PCWP: pulmonary capillary wedge pressure
PDE5I: phosphodiesterase 5 inhibitor
SVR: systemic vascular resistance
WRF: worsening renal function
